# Transplanted Heart Complicated by Intracardiac Aspergilloma With Pericardial Involvement

**DOI:** 10.1016/j.jaccas.2024.102574

**Published:** 2024-10-02

**Authors:** Saed Alnaimat, Alexis Arrigo, Srijana Maharjan, Joshua Longinow, Sunita Mahabir, Bang Tang, Masaki Tsukashita, Rasha Abdulmassih, George Bchech, Mithun Chakravarthy, Hayah Kassis-George, Amresh Raina

**Affiliations:** a Department of Cardiology, Allegheny General Hospital, Pittsburgh, Pennsylvania, USA; b Department of Internal Medicine, Allegheny General Hospital, Pittsburgh, Pennsylvania, USA; c Department of Pathology, Allegheny General Hospital, Pittsburgh, Pennsylvania, USA; d Department of Cardiothoracic Surgery, Allegheny General Hospital, Pittsburgh, Pennsylvania, USA; e Division of Infectious Disease, Allegheny General Hospital, Pittsburgh, Pennsylvania, USA

**Keywords:** aspergillosis, cardiac transplant, CMR, echocardiogram, endophthalmitis, fungal pericarditis, LV mass, mobile echodensity, multimodality imaging

## Abstract

A cardiac transplant recipient initially presented with right eye pain and blurry vision. After extensive workup, she was diagnosed with intracardiac aspergilloma with pericardial involvement complicated by endogenous endophthalmitis and acute myocardial infarction, treated in part with mechanical debulking using the Angiovac system (AngioDynamics).

## Graphical Abstract

**Figure:**
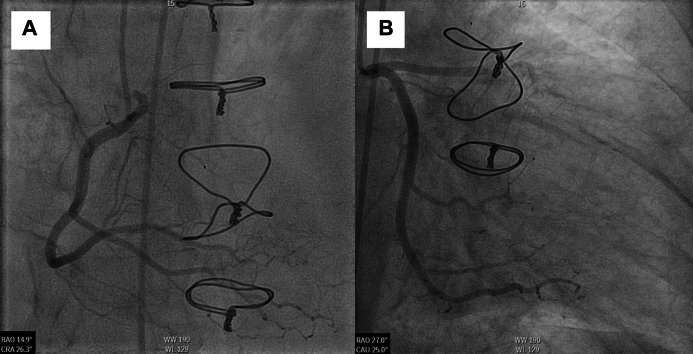

Fungal cardiac infections are extremely rare and opportunistic in nature. Only 1.3% to 6% of infective endocarditis cases are fungal in etiology. The majority of fungal endocarditis is caused by *Candida* species, whereas *Aspergillus* species are identified in 18% of cases.^1^ *Aspergillus* is a common filamentous mold that is abundant in the environment and is easily inhaled; however, healthy individuals have a natural immunity to *Aspergillus* spores.^2^ Fungal cardiac infections are usually associated with long-term use of indwelling catheters, posttransplant, and other immunocompromised states.^3^ These infections may present as myocarditis, endocarditis, intramyocardial abscess, or an intracardiac fungal mass (fungoma). Invasive aspergillosis with cardiac involvement is not only rare but poses a diagnostic challenge, and it has very poor prognosis.^4^

**Take-Home Messages**

• Patients with cardiac transplant may experience numerous possible complications, including opportunistic infections such as cardiac aspergillosis.
• Cardiac aspergillosis has various presentations, including intracardiac mass and pericardial effusion.
• Diagnosis and management of cardiac aspergillosis are challenging and entail a high index of suspicion, a multidisciplinary approach, and contemporary imaging and interventional techniques.

## History of Presentation

A 51-year-old woman had a late presentation of large anterior wall ST-segment elevation myocardial infarction complicated by cardiogenic shock. She underwent percutaneous coronary intervention to the left anterior descending artery. Subsequently, she required HeartMate 3 (Abbott) left ventricular assist device implantation 2 months after ST-segment elevation myocardial infarction because of end-stage ischemic cardiomyopathy with a severely reduced left ventricular ejection fraction (LVEF) of 10%. One year later, she underwent orthotopic heart transplant. She had a low rejection risk and was started on appropriate immunosuppression with tacrolimus (goal: 10-12 ng/mL), mycophenolate 1,000 mg twice daily, and a standard tapering course of prednisone. She was also maintained on microbiologic prophylaxis with sulfamethoxazole/trimethoprim 800/160 mg 3 times weekly, valganciclovir 900 mg daily, and clotrimazole 10 mg 3 times daily. She had several endomyocardial biopsies that showed no signs of rejection.

Approximately 6 months post-transplantation, the patient presented with acute-onset loss of vision in the right eye. Physical examination findings were significant for tachycardia with regular rhythm, normal jugular venous pressure, with normal cardiac auscultation. Dedicated ophthalmologic examination revealed a normal right eye intraocular pressure of 14 mm Hg. Anterior segment examination revealed mild conjunctival injection. Pupils were symmetric, equally round, and reactive, with no relative afferent pupillary defects. Dilated fundus examination revealed features of vitritis and retinal edema. Optic disc appeared normal. Her neurologic examination findings were otherwise normal.

## Past Medical History

Past medical history was significant for diabetes mellitus and coronary artery disease resulting in end-stage ischemic cardiomyopathy.

## Investigations

**Figure 1:**
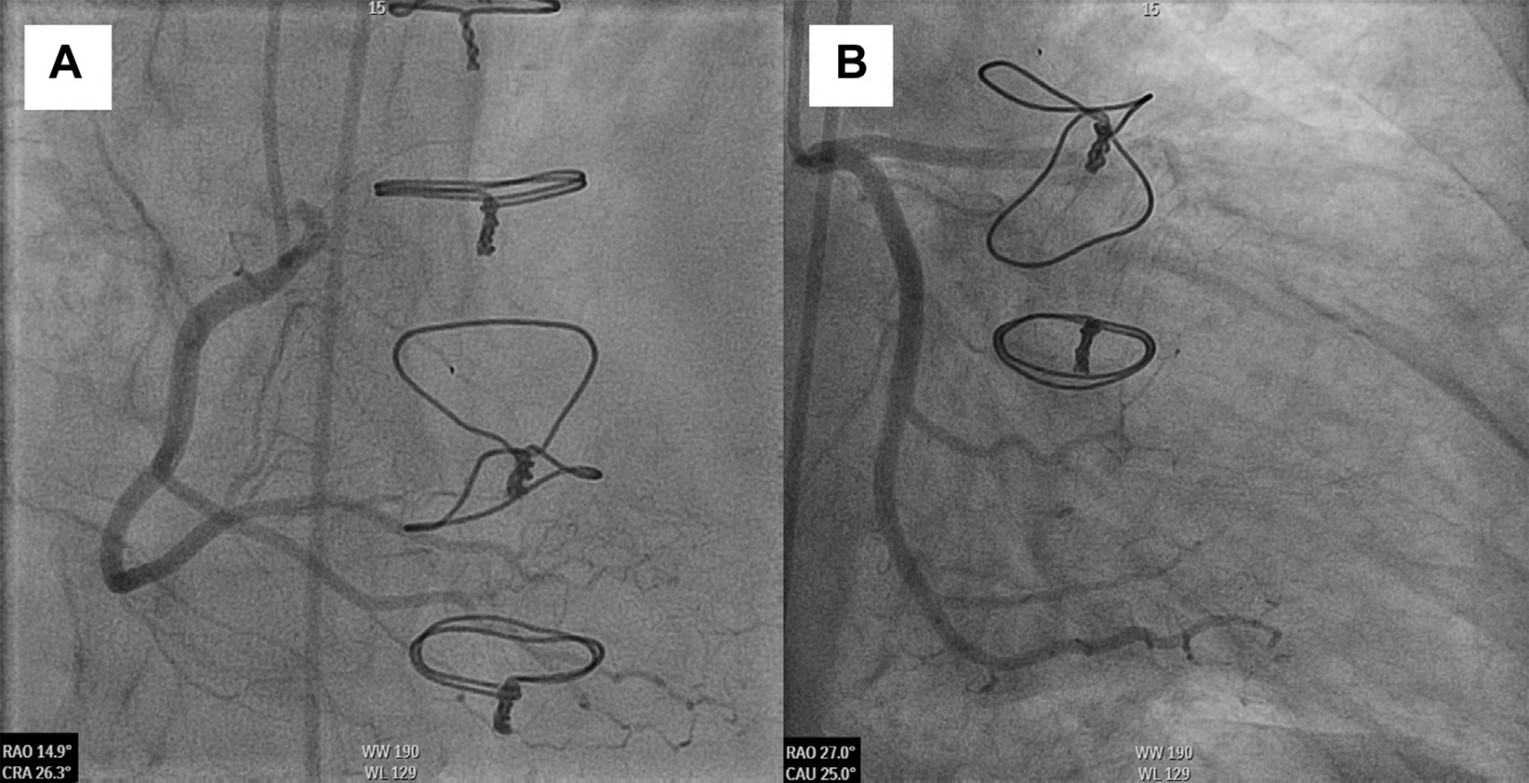
Coronary angiogram of the transplanted heart Coronary angiogram showing (A) normal right coronary system but (B) 100% occluded mid left anterior descending artery.

A contrast-enhanced transthoracic echocardiogram showed an LVEF of 42% with apical akinesis. A highly mobile intraventricular mass (2.1 × 1.3 cm) originating from the midanterior wall was concerning for a thrombus vs possible vegetation (Video 1, Video 2, Video 3, Video 4). Transthoracic echocardiogram also showed moderate mitral regurgitation and moderate tricuspid regurgitation, with no valvular vegetations noted. Cardiac catheterization showed 100% occlusion of the mid left anterior descending artery with subtotal occlusion of the mid diagonal branch, which was treated with percutaneous coronary intervention (Figure 1), although cardiac catheterization 5 months prior showed angiographically normal coronary arteries of the transplanted heart. Brain imaging findings were negative for acute intracranial abnormality, large vessel occlusion, or high-grade intracranial or extracranial stenosis. Computed tomography (CT) of the chest demonstrated bilateral pleural effusions without pulmonary consolidation or masses.

**Figure 2:**
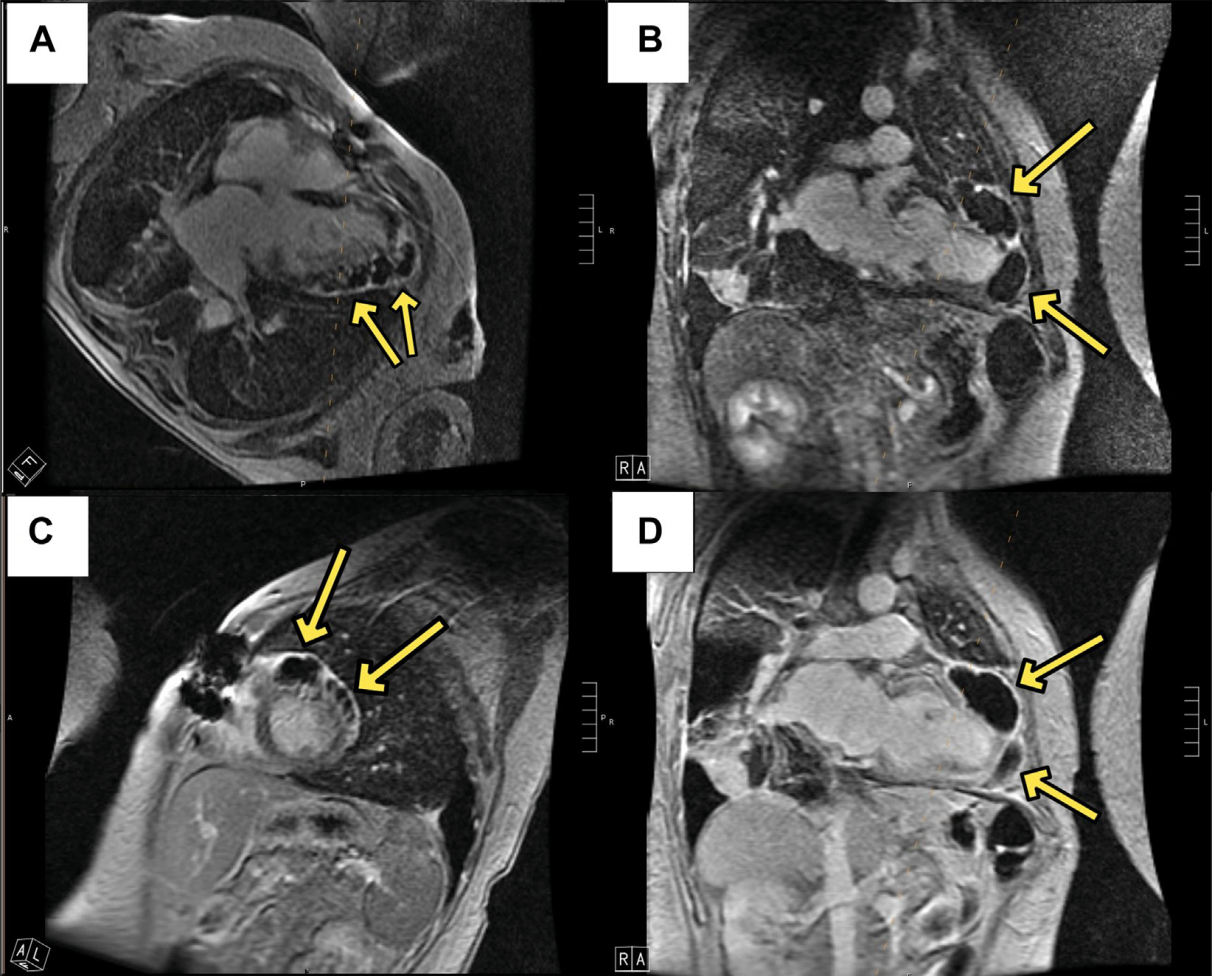
Cardiac Magnetic Resonance Late Gadolinium Enhancement Sequence Showing Multiple Loculated Pericardial Cavities Arrows denote extensive pericardial cavities with hyperenhancing rims invading epicardium. The absence of gadolinium contrast filling into these cavities indicates lack of direct communication with the intraventricular space. (A) The 4-chamber view. (B) The 2-chamber view. (C) The short-axis view. (D) The 2-chamber view with a long time to inversion of 600 ms.

The patient was started on anticoagulation for presumed LV thrombus. Although blood culture results remained negative, her funduscopic examination revealed vitritis, leading the ophthalmology team to suspect fungal etiology. Consequently, fungal serologies were conducted, revealing an elevated *Aspergillus* galactomannan. Cardiac magnetic resonance (CMR) showed an LVEF of 29% and redemonstrated a mobile mass attached to the midanterior wall. Additionally, CMR demonstrated multiple loculated pericardial cavities invading epicardium (the largest measuring 66 × 63 × 26 mm). These cavities were adherent to the LV wall and had hyperenhancing rims, suggesting inflammation. The cavities remained black on a long time-to-inversion sequence, suggesting they might contain coagulated material. It was difficult to evaluate for evidence of apical infarction given epicardial invasion and suboptimal images. However, the LV apex was dyskinetic, and distal segments were severely hypokinetic (Figure 2, Video 5, Video 6, Video 7, Video 8).

## Differential Diagnosis

The differential diagnosis of an intraventricular mass includes thrombus (the most common intracardiac mass), vegetation (infective or noninfective), benign tumor (myxoma, lipoma), malignant tumor (metastatic or primary such as sarcoma), healed vegetations, or abscesses.^5^ In our case, cardiac aspergilloma was the most likely differential diagnosis, given the underlying immunosuppression, positive galactomannan antigen, unexplained endophthalmitis/vitritis, and acute myocardial infarction. Extensive workup failed to reveal her original source of infection. She showed no evidence of pulmonary aspergillosis, sternal wound infection, or osteomyelitis. Although her blood culture results were negative for *Aspergillus*, transient fungemia remains the most likely etiology for her disseminated infection. The infection could have been inoculated at the time of transplant surgery. However, fungal air sampling conducted after identifying her case did not detect *Aspergillus* species in numerous samples collected from the transplant operating room, admission room, and cardiac catheterization laboratory.

## Management

The patient underwent surgical evacuation and washout of the pericardial effusion and pericardiectomy. To minimize operative risk, the procedure was performed via left thoracotomy approach. Intraoperatively, she was found to have dense adhesions between the pericardium and ribs. The pericardium was thickened and contained purulent material firmly attached to the myocardium. Pericardial tissue and fluid cultures grew *Aspergillus fumigate.* The patient was treated with systemic voriconazole for a minimum of 6 months, followed by lifelong suppressive therapy. Voriconazole levels were closely monitored, with values ranging between 2 and 5 μg/mL. She received intravitreal injection of voriconazole (0.1 mg) and was given prednisolone eye drops (1% ophthalmic suspension 4 times daily). Mycophenolate was discontinued, and she was kept on monotherapy immunosuppression with tacrolimus with a reduced goal of 8 to 10 ng/mL.

## Outcome and Follow-Up

**Figure 3:**
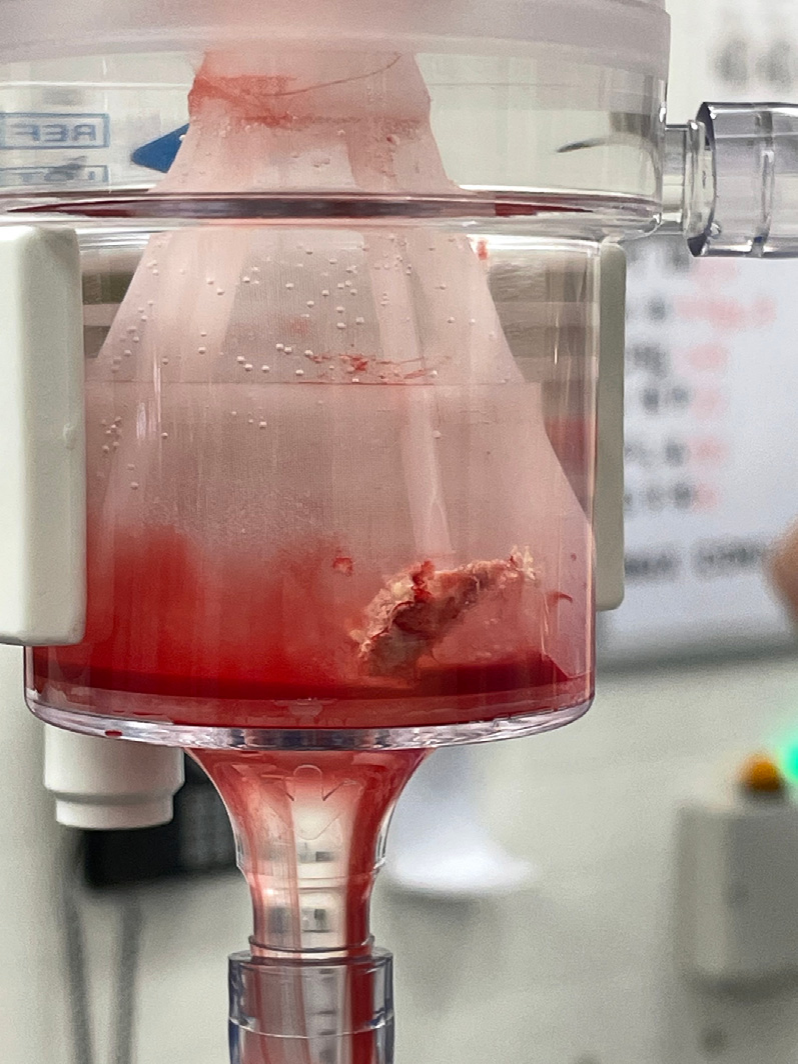
Gross Specimen of the Left Ventricular Mass Aspirated Via a Transcatheter Approach

**Figure 4:**
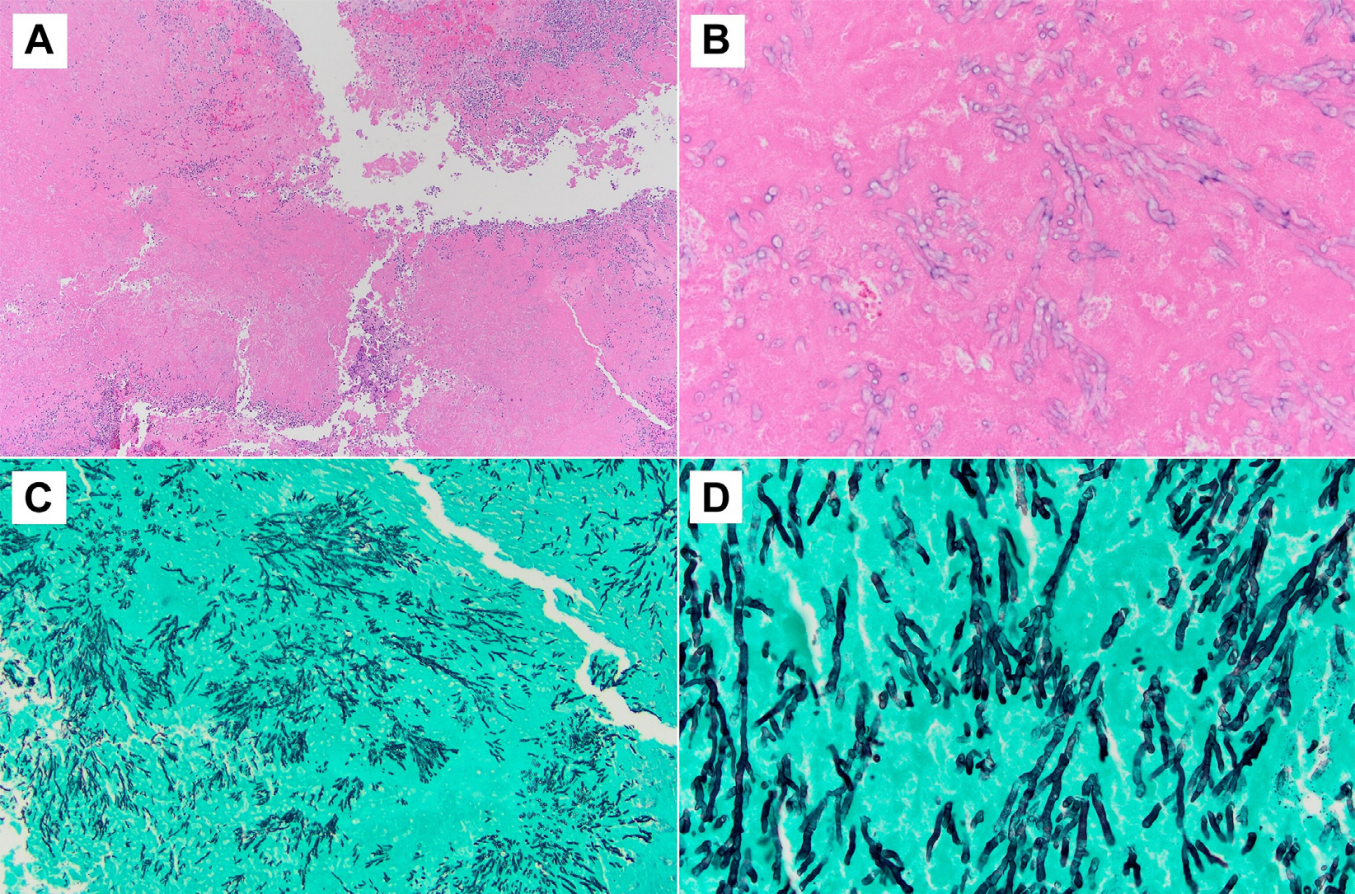
Hematoxylin and Eosin Slides Showing Fibrinous Material With Inflammatory Cells Higher-power zoom showing hyphae: (A) original magnification ×4 and (B) original magnification ×40. Grocott methenamine silver stain showing fungal hyphae with acute angle branching, consistent with *Aspergillus* species: (C) original magnification ×4 and (D) original magnification ×40.

**Figure 5:**
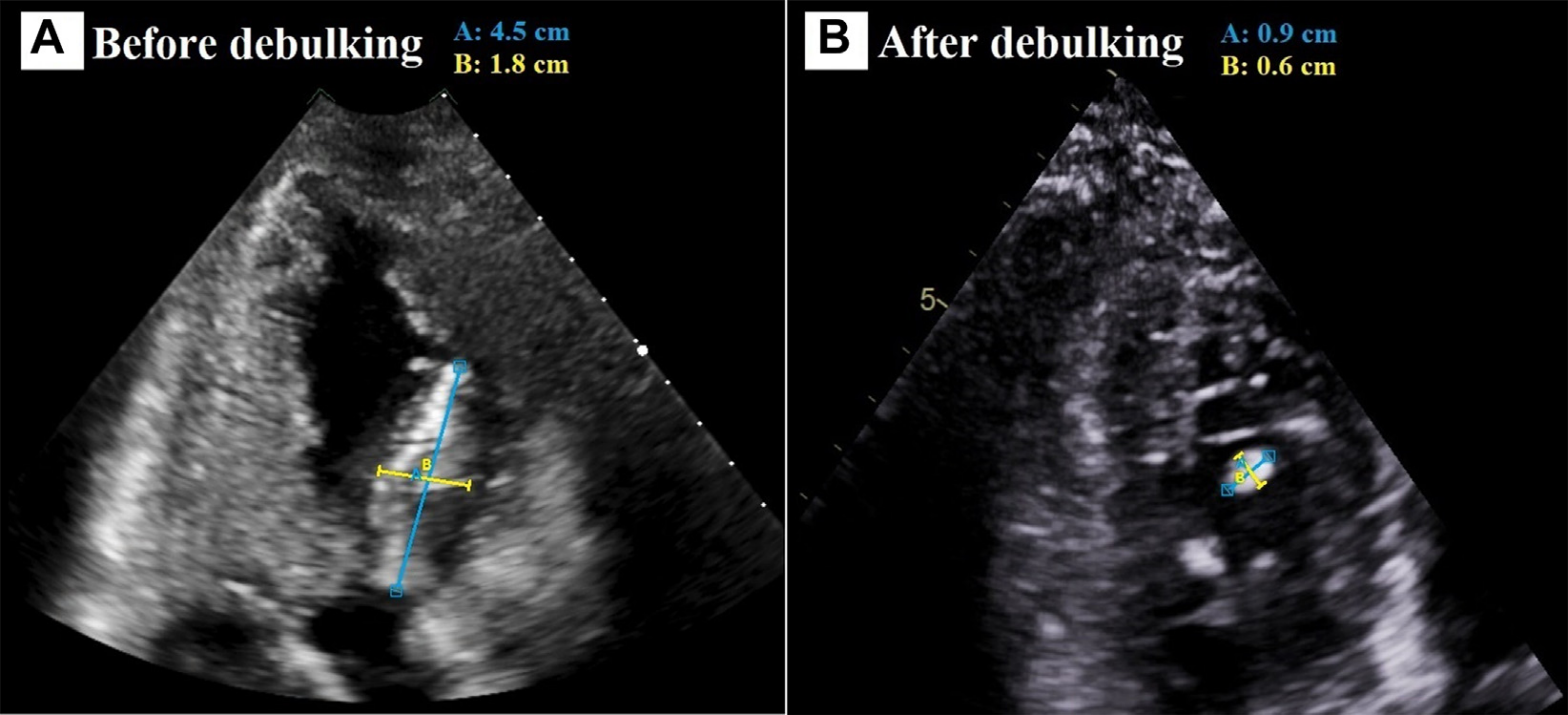
Echocardiogram in the Apical 2-Chamber View (Still Images) Demonstrating Successful Debulking of the Intracardiac Mass (A) Large intracardiac mass measuring 4.5 × 1.8 cm (before debulking). (B) Significant reduction of the intracardiac mass size to 0.9 × 0.6 cm (after debulking).

Despite these measures and antifungal treatment, the intracardiac mass was noted to be enlarging and more freely mobile, with concern for risk of further embolization (Video 9). After multidisciplinary discussion with the cardiac surgery, heart failure cardiology, and infectious disease teams, the LV mass was successfully aspirated via a transseptal approach using a 22-F AngioVac system (Figure 3). Histopathologic examination of the mass was consistent with *Aspergillus* species (Figure 4). She tolerated the procedure without any neurologic or hemodynamic compromise, and only a small residual stalk of the lesion remained (Figure 5). She was interviewed 1 week later. Although her right eyesight had not completely returned, she denied cardiac symptoms and has not had any other embolic complications.

## Discussion

Although invasive pulmonary aspergillosis is a well described entity in immunocompromised patients, cardiac involvement by *Aspergillus* is extremely uncommon and often fatal.^4^ There are 4 types of cardiac involvement by *Aspergillus*: intracavitary mass (aspergilloma); intramyocardial abscesses with variable degrees of invasion to endocardium and pericardium; valvular endocarditis (prosthetic or native); and, lastly, *Aspergillus* embolization causing myocardial infarction.^4^ Risk factors for cardiac aspergillosis include previous valve surgery, antibiotic use, immunosuppression, and malignancy.^6^ In a series of 19 published cases of intracavitary aspergilloma, 3 patients had solid organ transplants (lung, liver, and renal), and 3 patients with hematologic malignancies had stem cell transplants.^6^ Intracavitary aspergilloma has been previously reported in a cardiac transplant recipient who had a large (3.5 × 3.3 cm) right atrial mass.^7^ To our knowledge, there are no reported cases of intraventricular aspergilloma in cardiac transplant recipients in the literature.

Diagnosis of cardiac aspergillosis is challenging and often requires a high degree of clinical suspicion in combination with serologic evidence, advanced imaging techniques, and even invasive procedures for histopathologic confirmation. Blood cultures for detection of aspergillosis are often negative.^6^ Various CMR sequences enable tissue characterization and identification of intracardiac masses in locations where other imaging modalities may fail to identify (such as intramural or certain pericardial locations). This may potentially allow for earlier diagnosis in immunocompromised patients, resulting in improved prognosis. Echocardiogram plays an important role in the initial evaluation of cardiac functions and valvular pathology, and CT scan is an excellent tool to evaluate for extracardiac dissemination of aspergillosis. However, both echocardiogram and CT lack tissue characterization capabilities and may not be able to detect intramural masses.^4^ Fluorine-18-fluorodeoxyglucose positron emission tomography–CT may have an additive diagnostic value, especially in patients with prosthetic valve endocarditis.^6^

Treatment of intracardiac aspergillosis requires a combination of surgical intervention and antifungal therapy. The type of surgical intervention depends on the location and size of the aspergilloma. In our case, surgical removal of LV fungoma was not performed because it requires redo sternotomy and cardiopulmonary bypass, which carry a high risk of perioperative morbidity and mortality. Transcatheter aspiration of accessible intracardiac aspergillomas is feasible and, in this case, proved to be safe without major periprocedural morbidity. Antifungal therapy is continued for an extended duration (possibly lifelong) with either voriconazole or liposomal amphotericin B.^6^ Voriconazole is associated with better outcomes compared to amphotericin, including for patients with renal dysfunction.^8^ Isavuconazule is an alternative option; however, because of limited capabilities for drug monitoring, it would be less ideal in the context of acute illness.^9^ Although combination therapy with amphotericin has been reported in prior case reports, its use remains controversial. Interruption of antifungal therapy may lead to recurrence of such a virulent disease. Therefore, these patients require strict clinical and imaging follow-up as well as a multidisciplinary approach.

## Conclusions

We experienced a rare case of a cardiac transplant recipient with no prior history of pulmonary aspergillosis who developed an intraventricular and invasive pericardial aspergillosis. This was complicated by endogenous endophthalmitis and acute myocardial infarction, both of which are believed to represent embolic phenomena. Herein, we illustrate magnetic resonance features as well as the feasibility of transcatheter aspiration of intracardiac aspergilloma.

## Funding Support and Author Disclosures

The authors have reported that they have no relationships relevant to the contents of this paper to disclose.

## Appendix

Video 1 Transthoracic Echocardiogram, 4-Chamber View, Showing a Reduced LVEF, Apical Akinesis, and Highly Mobile Intracardiac Mass

Video 2 Transthoracic Echocardiogram, 2-Chamber View, Showing a Highly Mobile Intracardiac Mass Attached to the Midanterior Wall

Video 3 Transthoracic Echocardiogram, Short-Axis View, Redemonstrating the Intracardiac Mass

Video 4 Contrast-Enhanced Transthoracic Echocardiogram, 4-Chamber View, Redemonstrating the Highly Mobile Intracardiac Mass and Apical Akinesis

Video 5 Steady-State Free Precession CMR, 2-Chamber View, Showing 2 Large Cavities Surrounding the Left Ventricle Apex and Anterior Wall Note the highly mobile intracardiac mass.

Video 6 Steady-State Free Precession CMR, 4-Chamber View, Redemonstrating Pericardial Cavities Note bilateral pleural effusions, mitral regurgitation, and tricuspid regurgitation jets.

Video 7 Steady-State Free Precession CMR, 3-Chamber View, Redemonstrating the Large Cavity Surrounding the Left Ventricle Apex Note apical dyskinesis.

Video 8 CMR Spatial Modulation of Magnetization Tagging Sequence Showing Epicardial Cavities Adherent to the Left Ventricle Wall

Video 9 Transthoracic Echocardiogram, 4-Chamber View, Showing Enlarging and Highly Mobile Intracardiac Mass
